# Comprehensive identification of virulence factors required for respiratory melioidosis using Tn-seq mutagenesis

**DOI:** 10.3389/fcimb.2015.00078

**Published:** 2015-11-04

**Authors:** Maria G. Gutierrez, Deborah R. Yoder-Himes, Jonathan M. Warawa

**Affiliations:** ^1^Department of Microbiology and Immunology, University of LouisvilleLouisville, KY, USA; ^2^Department of Biology, University of LouisvilleLouisville, KY, USA; ^3^Center for Predictive Medicine, University of LouisvilleLouisville, KY, USA

**Keywords:** respiratory melioidosis, intubation-mediated intratracheal (IMIT) inoculation, mouse infection models, Tn-seq, type 3 secretion, type 6 secretion, capsular polysaccharide

## Abstract

Respiratory melioidosis is a disease presentation of the biodefense pathogen, *Burkholderia pseudomallei*, which is frequently associated with a lethal septicemic spread of the bacteria. We have recently developed an improved respiratory melioidosis model to study the pathogenesis of *Burkholderia pseudomallei* in the lung (intubation-mediated intratracheal [IMIT] inoculation), which more closely models descriptions of human melioidosis, including prominent septicemic spread from the lung and reduced involvement of the upper respiratory tract. We previously demonstrated that the Type 3 Secretion System cluster 3 (T3SS3) is a critical virulence determinant for *B. pseudomallei* when delivered directly into the lung. We decided to comprehensively identify all virulence determinants required for respiratory melioidosis using the Tn-seq phenotypic screen, as well as to investigate which virulence determinants are required for dissemination to the liver and spleen. While previous studies have used Tn-seq to identify essential genes for *in vitro* cultured *B. pseudomallei*, this represents the first study to use Tn-seq to identify genes required for *in vivo* fitness. Consistent with our previous findings, we identified T3SS3 as the largest genetic cluster required for fitness in the lung. Furthermore, we identified capsular polysaccharide and Type 6 Secretion System cluster 5 (T6SS5) as the two additional major genetic clusters facilitating respiratory melioidosis. Importantly, Tn-seq did not identify additional, novel large genetic systems supporting respiratory melioidosis, although these studies identified additional small gene clusters that may also play crucial roles in lung fitness. Interestingly, other previously identified virulence determinants do not appear to be required for lung fitness, such as lipopolysaccharide. The role of T3SS3, capsule, and T6SS5 in lung fitness was validated by competition studies, but only T3SS3 was found to be important for respiratory melioidosis when delivered as a single strain challenge, suggesting that competition studies may provide a higher resolution analysis of fitness factors in the lung. The use of Tn-seq phenotypic screening also provided key insights into the selective pressure encountered in the liver.

## Introduction

*Burkholderia pseudomallei*, the etiological agent of melioidosis, is a motile Gram-negative bacterium classified as a Tier 1 Select Agent. While endemic to Southeast Asia and northern Australia (Cheng and Currie, 2005), an increasing number of melioidosis cases have been reported in Latin America (Inglis et al., 2006), the Caribbean (Wiersinga et al., 2006), Africa (Schweizer et al., 2014) and other tropical regions worldwide (Cheng and Currie, 2005). The bacterium is an environmental saprophyte generally found in the soil and water reservoirs in indigenous areas (Galyov et al., 2010) where it opportunistically infects an array of plant (Kaestli et al., 2012), animal, and human hosts (Cheng and Currie, 2005). The most common routes of infection in humans include percutaneous inoculation, ingestion, or inhalation of the bacterium from the environment (Brett and Woods, 2000; Wiersinga et al., 2006; Lazar Adler et al., 2009). Melioidosis manifestations may include asymptomatic seroconversion, febrile illness, pneumonia, hepatic and splenic abscesses, chronic melioidosis and sepsis syndrome (Holden et al., 2004; Wiersinga et al., 2006; Cheng et al., 2013). Risk factors associated with melioidosis include diabetes mellitus, alcoholism, renal failure, and chronic pulmonary disease (Wiersinga et al., 2006).

Respiratory melioidosis is a disease presentation of high relevance to both naturally-occurring and biodefense-related studies; indeed, *B. pseudomallei* is lung tropic even in cases of percutaneous inoculation (Currie et al., 2000). Both our lab and others have previously shown that the nasal cavity represents the predominant site of *B. pseudomallei* colonization in intranasally-inoculated mice (Owen et al., 2009; Warawa et al., 2011), whereas direct lung instillation of bacteria can abrogate nasal cavity colonization and the associated central nervous system involvement (Revelli et al., 2012; Gutierrez et al., 2015). We have further characterized that lung-specific instillation of *B. pseudomallei* results in a shift of moribund endpoint from a predominant nasal cavity infection in the intranasal model to a greater bacterial proliferation in the lung and disseminated spread (Gutierrez et al., 2015), more closely resembling descriptions of human melioidosis. Furthermore, the intubation-mediated intratracheal (IMIT) inoculation method has facilitated the discovery of a spread-deficiency phenotype for a capsular polysaccharide mutant from the lung (Gutierrez et al., 2015), which had not been discovered in mice succumbing to nasal cavity colonization (Warawa et al., 2011), and yet meets the predicted role for capsule in mediating protection from complement during dissemination (Reckseidler-Zenteno et al., 2005). Thus, the IMIT lung-specific melioidosis model has begun to provide unique insights into the roles of *B. pseudomallei* virulence determinants that have not been identified in other respiratory melioidosis models. We therefore sought to identify the roles for additional *B. pseudomallei* virulence determinants using the IMIT model of lung-specific disease.

Tn-seq is a powerful new tool used to identify a full repertoire or genes required for an organism's viability in a selective environment by combining saturation mutagenesis and Next Generation Sequencing (Van Opijnen et al., 2009). Recent Tn-seq studies identified essential genes required to support *in vitro* growth for the *Burkholderia pseudomallei* K96243 strain (Moule et al., 2014) and the closely related *Burkholderia thailandensis* E264 strain (Baugh et al., 2013; Gallagher et al., 2013). These studies identified potential antimicrobial drug targets in key constituents of metabolic pathways, cell structure and genes required for nucleotide and amino acid synthesis, and further estimated that ~8% of the *Burkholderia* genome represent essential genes (Baugh et al., 2013; Moule et al., 2014). Importantly, Tn-seq has not previously been used to identify *B. pseudomallei* genes required to support growth in the selective pressure of mammalian host tissues. In the present study, we investigated the potential of Tn-seq to identify virulence determinants required by *B. pseudomallei* to colonize mammalian lungs in a mouse model of respiratory melioidosis as well as to disseminate to the liver and spleen. These studies take advantage of our IMIT respiratory melioidosis model to non-invasively target delivery of a Tn-seq library directly into the lungs of mice to specifically identify genes supporting pulmonary disease in the absence of potential interplay between upper and lower respiratory tract infections.

## Materials and methods

### Bacterial strains and culture

*Burkholderia pseudomallei* and *Escherichia coli* strains (Table 1) were routinely cultured in Lennox Broth (LB) at 37°C. For inoculum preparation, *B. pseudomallei* strains were subcultured 1:25 in dialyzed and chelated Trypticase Soy Broth (TSBDC) supplemented with 50 μM monosodium glutamate from overnight LB cultures and grown for 3 h at 37° C. Antibiotics were used at the following concentrations when appropriate: kanamycin (Km), 25 μg/mL; polymyxin B (Pm), 50 μg/mL; streptomycin (Sm), 100 μg/mL; gentamicin (Gm), 20 μg/mL.

**Table 1:** **Strains and plasmids used in this study**.

**Strain**	**Description**	**References**
S17−1	*E. coli* conjugation strain, λpir	Simon et al., 1983
S17−1/pKAS46−*ara*P_tolC_*lux*	Bioluminescence allelic exchange construct	Warawa et al., 2011
S17−1/pKAS46−Δ*tssC−5*	T6SS5 mutant allelic exchange construct	This study
DD503	*B. pseudomallei* 1026b derivative, Pm^R^ Sm^R^ Km^S^ Gm^S^, lacking AmrAB-OprA efflux pump	Moore et al., 1999
DD503 *ΔtssC−5*	T6SS5 mutant	This study
DD503 *ΔsctU_*Bp*3_*	T3SS3 mutant	Warawa and Woods, 2005
JW270	DD503 Δ*wcb* capsule mutant	Warawa et al., 2009
JW280	DD503::P_tolC_-*luxCDABE*	Warawa et al., 2011
JW280 *ΔtssC−5*	Bioluminescent T6SS5 mutant	This study
MGBP001	DD503::P_tolC_-*luxCDABE*-Gm	This study
MGBP001 *ΔtssC-5*	Bioluminescent, Gm^R^ T6SS5 mutant	This study
MGBP001 *ΔsctU_*Bp*3_*	Bioluminescent, Gm^R^ T3SS3 mutant	This study
MGBP001 Δ*wcb*	Bioluminescent, Gm^R^ capsule mutant	This study
**Plasmid**		
pBTK30	Vector containing *Himar1* C9 transposase	Goodman et al., 2004
pSAM-Bt	Tn-seq vector for use in *Bacteroides thetaiotaomicron*	Goodman et al., 2009
pSAM-DYH	pSAM_Bt with the transposase from pBTK30 inserted as a *Bam*HI fragment to replace the native transposase and promoter	Skurnik et al., 2013a
pEXKm5	Gene replacement vector for *Burkholderia* species	López et al., 2009
pSAM-DKm	Tn-seq vector	This study
pKAS46	Allelic exchange vector providing Km^R^ and Sm^S^ in DD503 genetic background	Skorupski and Taylor, 1996
pGSV4	Promoterless bioreporter vector harboring *luxCDABE* operon	Gutierrez et al., 2015
pCR®-Blunt II-TOPO	Topoisomerase-conjugated cloning vector	Invitrogen
pUC19	Cloning vector	New England Biolabs

### Tn-seq library preparation

Plasmids used in this study are identified in Table 1. To construct the transposon delivery vector pSAM-DKm, the *mariner*-family transposon, *Himar1* C9 transposase and its upstream regulatory region were PCR amplified from pBTK30 and cloned as a BamHI restriction fragment into pSAM-Bt to yield pSAM-DYH. The kanamycin resistance cassette from pEXKm5 was PCR amplified and inserted into the MfeI and XbaI restriction sites in place of the erythromycin resistance cassette and the resulting plasmid, pSAM-DKm was verified by PCR. To generate the *B. pseudomallei* Tn-seq library, the *E. coli* S17-1 strain transformed with pSAM-DKm was conjugated with the luminescent *B. pseudomallei* parent strain JW280 for 2 h at 37°C. Transposon mutants were selected for on PmKm LB agar plates and grown at 37°C for 24 h. A library of 2 × 10^4^ mutants were pooled, grown in PmKm LB broth with shaking at 37°C and cryopreserved.

### Ethics and biosafety statement

All animal studies were conducted under Biosafety Level 3 conditions using 8–10 week old female albino C57BL/6J mice (B6 (Cg)-Tyr^c−2J^/J, Jackson Laboratories) bred at the University of Louisville (Protocol Number 11113). These studies were approved by the University of Louisville Institutional Animal Care and Use Committee (Protocol numbers 10073 and 13053) in agreement with NIH guidelines and the “Guide for the Care and Use of Laboratory Animals” (NRC). University of Louisville is approved for use of the Tier 1 select agent *B. pseudomallei* with continuous registration from the Centers for Disease Control since 2010.

### Tn-seq screen

A *B. pseudomallei* Tn-seq library TSBDC culture was washed into phosphate buffered saline (PBS) and OD_600_ readings were used to estimate bacterial concentrations in order to prepare an inoculum of 10^4.74^ CFU/50 μL. A group of three mice were inoculated with 50 μL of bacterial suspension of Tn-seq library by intubation mediated intratracheal (IMIT) instillation, as described (Lawrenz et al., 2014). Disease progression was monitored twice daily by optical diagnostic imaging of bioluminescent *B. pseudomallei* until study completion, as described (Gutierrez et al., 2015). Animals were euthanized at moribund endpoints (66 h post-infection) and necropsied to collect lungs, liver and spleen. Tissues were homogenized and bacteria from these tissues were cultured in LB broth overnight at 37°C with shaking. Bioluminescence imaging data was processed as described (Lawrenz et al., 2014).

Chromosomal DNA was purified from triplicate biological samples, as described (Beji et al., 1987), and pooled for each tissue. Genomic DNA was digested with the MmeI restriction enzyme overnight at 37°C, treated for 20 min at 80°C to inactivate the enzyme, purified using the QIAquick, PCR purification kit (Qiagen) according to the manufacturer's instructions, and concentrated with a speed vacuum to a final volume of 30 μL. Restricted fragments were separated by electrophoresis in a 1% agarose gel and fragments corresponding to 1.2–1.5 Kb were isolated using the QIAquick Gel Extraction Kit (Qiagen). Fragments were ligated to barcoded double stranded adaptors using T4 DNA ligase in 50 μL reactions overnight at 16°C, treated at 65°C for 10 min to inactivate the ligase, and purified using the QIAquick, PCR purification kit. Transposon-genome junctions were amplified by PCR using the LIB_PCR_5 (5′-CAAGC AGAAGACGGCATACGAAGACCGGGGACTTATCATCCAACC TGT-3′) and LIB_PCR_3 (5′-AATGA TACGGCGACCACCGAACACTCTTTCCCTACACGACGCTCTTCCGA TCT-3′) primers (Goodman et al., 2009) using HiFi DNA Polymerase (KAPA Biosystems). Confirmation of a 125 base pair product was conducted using 1% agarose gel electrophoresis, and libraries were sequeced using Illumina 50-bp single end sequencing at the Harvard Medical School Biopolymer Facility. Data was analyzed by sorting based on barcodes using a custom script in Java Eclipse, then using CLC Genomics Workbench v. 7.2 for subsequent bioinformatics. Reads were trimmed to remove adapter and transposon sequences and aligned to the *B. pseudomallei* 1026b genome using the default settings in the RNA-seq function in CLC. Reads that mapped to multiple locations were discarded. The resultant reads were counted and normalized to the reads per kilobase of transcript per million reads mapped (RPKM) and compared across sample sites. Significance was assessed using the On Proportions function of CLC using the Kals's test option and the Bonferroni multiple testing correction was applied to the comparisons. Initially, fold change measurements of less than −3 (reduced in the mouse samples compared to the input pool) with a Bonferroni-adjusted *p*-value of less than 0.05 were considered significant.

### Early dissemination studies

Early dissemination studies were conducted using the *B. pseudomallei* Tn-seq library strain to infect groups of three animals by IMIT as described above, euthanizing groups at 6, 12, and 24 h post-infection. Lung, liver and spleen from individual animals were collected at necropsy, homogenized and serial diluted for bacterial enumeration as described (Lawrenz et al., 2014).

### Type 6 secretion system mutagenesis

To generate a Type 6 Secretion System cluster 5 (T6SS5) mutant in *B. pseudomallei*, an internal in-frame 1437 bp region of the *tssC-5* gene was deleted. Briefly, flanking fragments in the 5′ and 3′ region of the *tssC-5* gene were amplified with the following primer sets: 5-tssC-F, GAAGAATTCGCGTAGAACAGCAGCAGCAGCAGCCCCGC; 5-tssC-R, GTCAAGCTTCGGGGATTGCAGGTGTTCGCCTTCCATGGTC; 3-tssC-F, GTCAAGCTTTCGCTCGTCGGCAAGCTCGAAAAGCGCTAGG; 3-tssC-R, GGCGGTACCTTGCGCGAAGCCGCCCGCGAG. The amplified 5′ *tssC-5* fragment was cloned into pSK as an EcoRI/HindIII fragment to yield pSK-5′tssC. The amplified 3′ *tssC-5* fragment was cloned into pSK-5′tssC as a HindIII/KpnI fragment to yield pSK-Δ*tssC-5*. The assembled fragment was cloned into pKAS46 as an EcoRI/KpnI fragment to yield pKAS46-Δ*tssC-5* and conjugated with the parent *B. pseudomallei* strain DD503. Finally, the bioluminescent strain JW280 Δ*tssC-5* was constructed by conjugation of DD503 Δ*tssC-5* with S17-1/pKAS46-*ara*P_tolC_*lux*, as described previously (Warawa et al., 2011).

### Virulence assessment of a T6SS5 mutant in respiratory melioidosis

Animal studies were conducted to compare the virulence of JW280 and the JW280 Δ*tssC-5* mutant by IMIT infection, as described above. Groups of five animals were euthanized at moribund end point defined as loss of righting reflex. Thus, we define LD_50_ in our studies as onset of moribund symptoms rather than to use death as an endpoint. Mice were necropsied and lung, liver and spleen were isolated and imaged for bioluminescence in 24-well black plates. The equations used to perform correlations between bioluminescent signal and colony forming units (CFU) have been previously defined (Gutierrez et al., 2015).

### Competition studies

A gentamicin resistance cassette was amplified with flanking NotI restriction sites from the pGSV4 vector (Gutierrez et al., 2015) with the primers Gm cass NotI(+) (GCGGCCGCAGATTTAAATTAATTAAGAGCTAGAATTGACATAAGCCTG) and Gm cass NotI(−) (GAAGCGGCCGCGGCGTTGTGACAATTTACCGAACAACTC). The Gm cassette was cloned into the pCR®-Blunt II-TOPO cloning vector (Invitrogen) and was then cloned into the pUC19-*ara*P_tolC_*lux* vector as a NotI/NotI fragment to yield pUC19-araP_tolC_*lux*-Gm. The *ara*P_tolC_*lux*-Gm fragment was cloned into pKAS46 to generate the pKAS46-*ara*P_tolC_*lux*-Gm and was transformed into S17-1 cells to generate the S17-1/pKAS46-*ara*P_tolC_*lux*-Gm strain. This strain was conjugated with the *B. pseudomallei* parent strain DD503, capsule polysaccharide mutant (JW270), Type 3 Secretion System translocation-deficient mutant (DD503 Δ*sctU*_*Bp*3_) and Type 6 Secretion System mutant (DD503 Δ*tssC-5*) to generate MGBP001, MGBP001 Δ*wcb*, MGBP001 Δ*sctU*_*Bp*3_, and MGBP001 Δ*tssC-5*, respectively, by allelic exchange as previously described (Warawa and Woods, 2005).

Competition studies were conducted by challenging animals by IMIT with ~10^4.0^ CFU of a 1:1 mixture of non-luminescent *B. pseudomallei* DD503 plus one of the four luminescent, gentamicin-resistant MGBP001 strains. The study was completed at 67 h post-infection at which point lung, liver and spleen were isolated and homogenized. Homogenates were plated in replicate on both LB and LB-Gm plates for bacterial enumeration and competitive index calculations.

### Statistical analysis

Two-Way ANOVA followed by the Bonferroni post-test, Student's *T*-test, Log-rank survival analysis (Mantel-Cox test and Gehan-Breslow-Wilcoxon test), One-Way ANOVA followed by Tukey's Multiple Comparison test were conducted using GraphPad Prism.

## Results

### Lung-specific mouse infection with the Tn-seq insertion library

**Figure 1:**
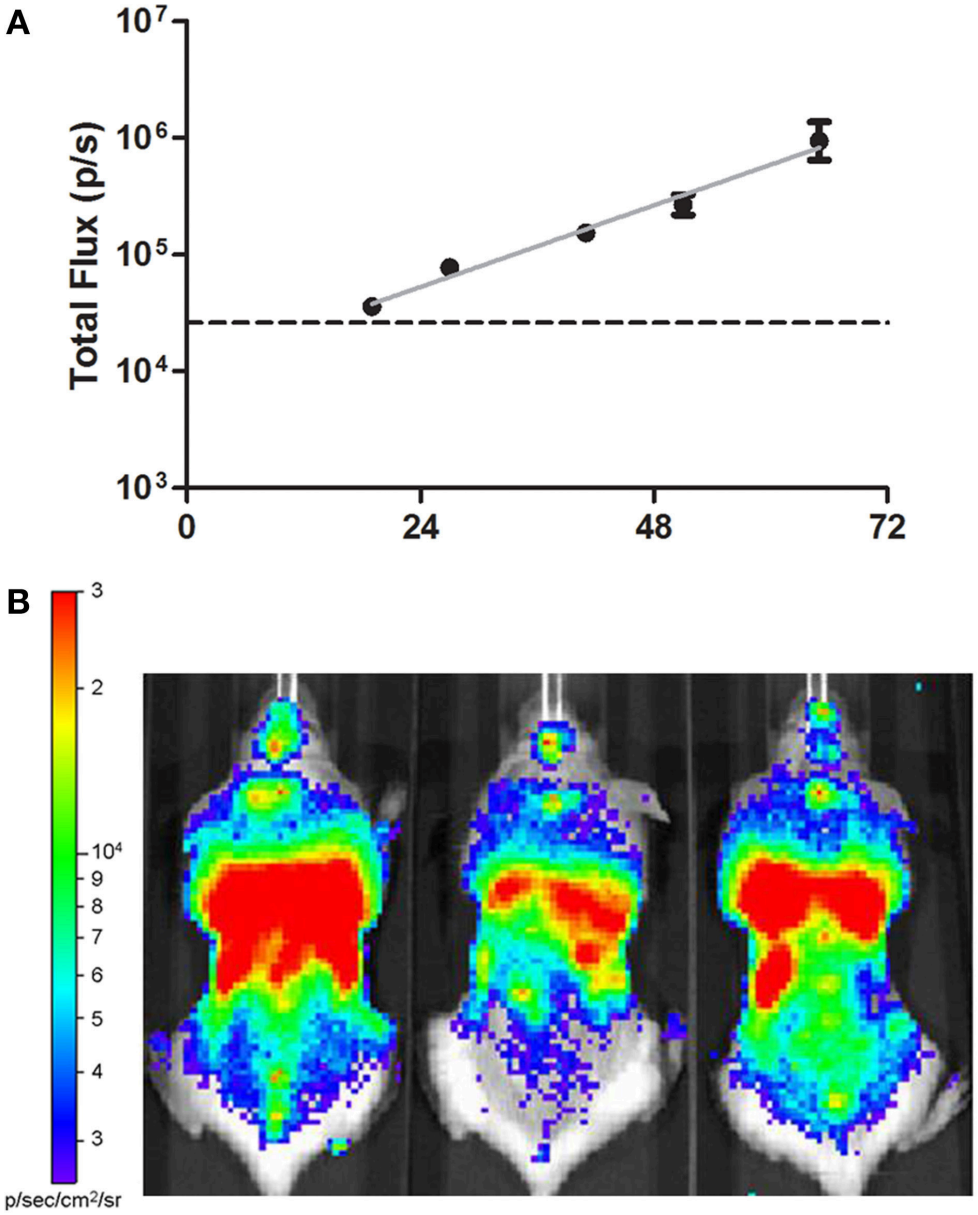
***In vivo* disease progression of *Burkholderia pseudomallei* Tn-seq transposon library**. **(A)** *In vivo* growth of the *B. pseudomallei* transposon library in the thoracic cavity of three C57BL/6J female albino mice infected by IMIT was monitored twice daily with an *in vivo* imaging system using luminescence as a read out for bacterial replication. Total luminescence was enumerated using a region of interest (ROI) centered on the thoracic cavity. The dashed line indicates technical 95% limit of detection. **(B)** Whole body imaging of moribund C57BL/6J mice infected with *B. pseudomallei* transposon library at 66 h post-infection. The logarithmic scale bar is provided for the standardized data presentation of 2.5 × 10^3^–3 × 10^4^ p/s/cm^2^/sr.

We sought to identify virulence determinants required by *B. pseudomallei* to cause disease specifically in the lung, as well as for subsequent disseminated spread to liver and spleen. Accordingly, we generated a transposon insertion library composed of 20,000 insertion mutants in the genome of the *B. pseudomallei* luminescent strain JW280, a derivative of the 1026b clinical strain. To select against transposon-inactivated genes required for lung colonization and systemic spread *in vivo*, we challenged pools of C57BL/6J albino female mice with 10^4.74^ CFU of a transposon insertion library. This inoculum was within 10 median lethal doses (LD_50_) of the JW280 strain (Gutierrez et al., 2015), and achieved ~4x coverage of the genome and 4x mutant strain representation in the challenge inoculum. We monitored the growth rate of the mutant library by bioluminescence starting at 19 h post-infection and observed that in the lungs, the library pool exhibited logarithmic growth at a rate consistent with our previously observed JW280 parent strain growth rate (Gutierrez et al., 2015; Figure 1A). By 66 h post-infection, all animals had reached moribund stage, characterized by acute respiratory infection and systemic spread (Figure 1B) and at this point all animals were euthanized and lungs, liver and spleen were isolated. These data indicate that *in vivo* disease progression in C57BL/6J mice infected with the transposon insertion library followed an acute course of infection comparable to that of the parent *B. pseudomallei* strain (Gutierrez et al., 2015).

### Transposon insertion sequencing and mapping

**Figure 2:**
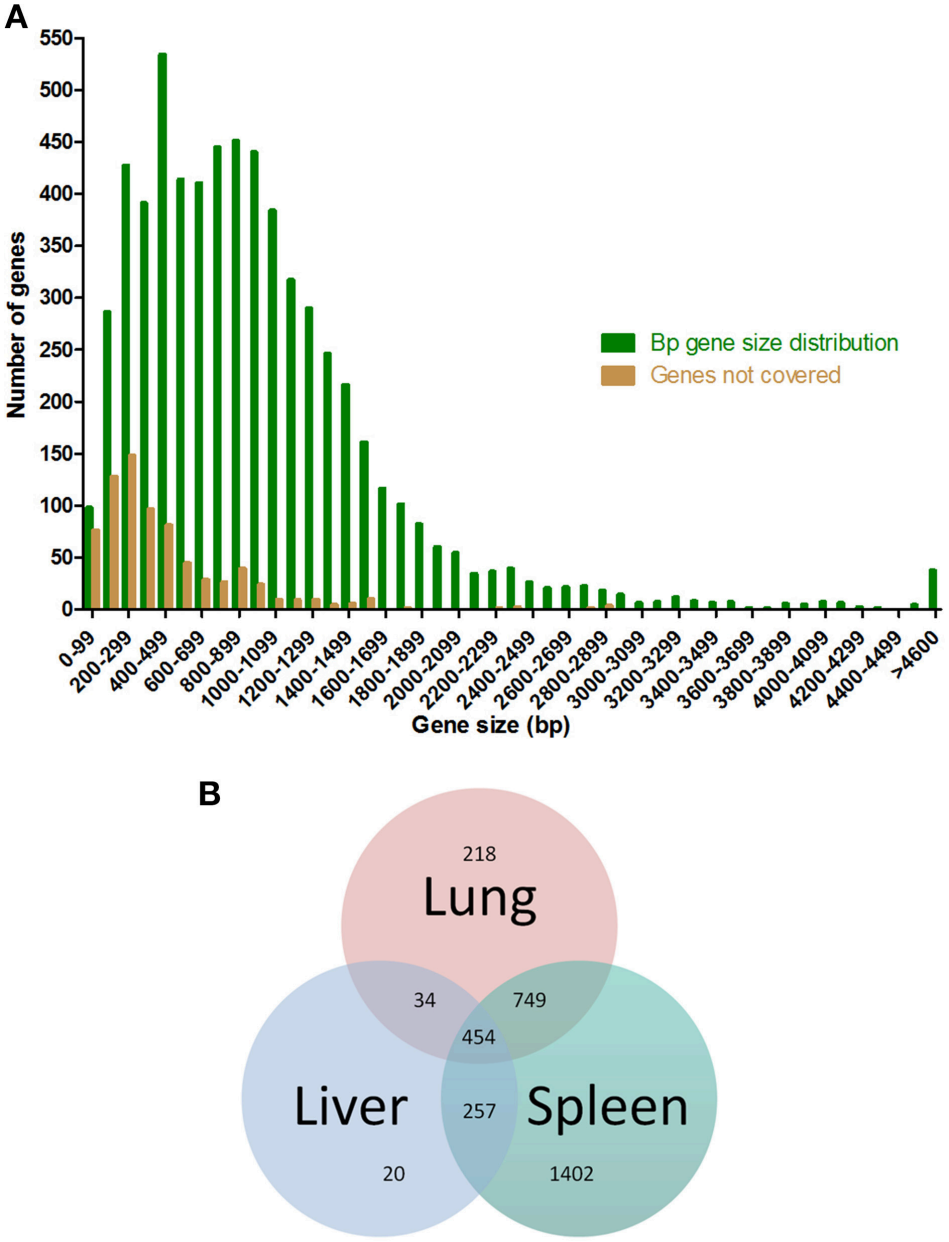
**Gene size distribution of the *Burkholderia pseudomallei* genome and transposon insertion coverage**. **(A)** Histogram of the *B. pseudomallei* genome (green bars) and percentage of genes lacking transposon insertions (brown bars), both binned at 100 bp gene size intervals. **(B)** Tn-seq data sets were analyzed for *B. pseudomallei* Tn-seq mutants lost by selective pressures in the lung (1455); liver (765); spleen (2862) at a 3-fold reduction in relative abundance relative to the input pool. Venn diagram illustrating mutants lost in the three tissues, with 454 genes being selected against in all three tissues.

We performed sequencing of the input transposon insertion library to determine transposon insertion density and assess coverage of the *B. pseudomallei* JW280 genome. Sequencing of the input mutant library mapped 1.1268 million reads to the *B. pseudomallei* 1026b genome and revealed that 88% percent of genes contained a transposon insertion. Approximately 8% of the *B. pseudomallei* genome is estimated to represent essential genes required for *in vitro* growth (Moule et al., 2014); thus only 92% of *B. pseudomallei* genes can be mutanted and our Tn-seq library approached full saturation. As expected, larger genes received better coverage efficiency, corresponding to the random nature of Marineer transposon insertion events favoring higher frequency insertion into larger genes (Figure 2A).

Tn-seq analyses typically investigate changes in library diversity between two conditions using a 2-fold cutoff criteria for very large libraries investigated in *in vitro* studies (Skurnik et al., 2013a,b; Wiles et al., 2013). However, we had limited the size of our Tn-seq library to achieve a biologically relevant infection and therefore we increased the stringency of our initial data analysis to a 3-fold cutoff to account for an increase in variation noise with a smaller library studied *in vivo*. Sequencing of the output libraries showed that at a 3-fold loss relative to the input library, transposon mutants in the lungs sustained selective pressure leading to clearance of 1455 transposon-inactivated genes required for lung colonization relative to the input pool (Figure 2B and Supplemental Table 1). We hypothesized that lung-specific delivery of the *B. pseudomallei* mutant library would sustain selective pressure in the lung, which would lead to an initial loss of library diversity and subsequent additional diversity loss following spread to the liver and spleen. As expected, the transposon library was further reduced in the spleen (2862 total genes lost relative to input pool), suggestive of loss of library diversity both in the lung and due to loss of genes required for disseminated spread. Surprisingly, we observed a greater retention of diversity of the transposon library in the liver relative to the lung (765 mutants lost in the liver compared to 1455 in the lung), suggesting that a representative library pool reached this site prior to effects of the selective pressure exhibited in the lung (Figure 2B). Further, the retention of library diversity in the liver relative to the lung suggests that the liver does not exhibit as strong a selective environment as the lung, and that the early spread to the liver is at high titer so as to avoid potential bottlenecking effects of spread from one tissue to another. Taken together, these data suggest that *B. pseudomallei* spread from the lung to the liver is a prominent dissemination path which occurs as an early event, resulting in a reduced selective pressure in the liver.

### *Burkholderia pseudomallei* early hepatic dissemination

**Figure 3:**
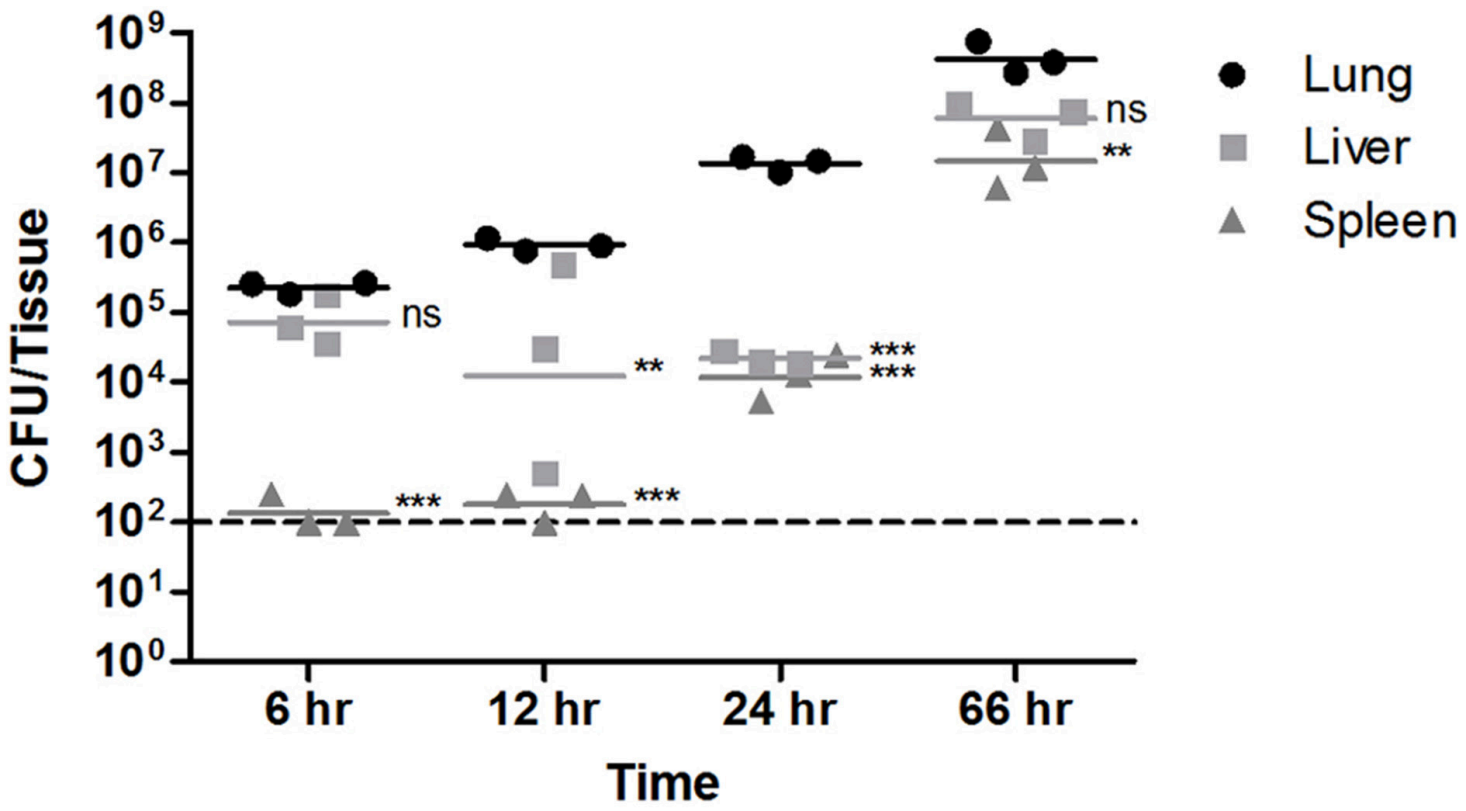
***Burkholderia pseudomallei* Tn-seq transposon library tissue burdens at key sites of infection**. Groups of three C57BL/6 female albino mice infected with the *B. pseudomallei* Tn-seq transposon library were euthanized at 6, 12, and 24 h post-infection. Bacterial titers in the lungs, liver and spleen were enumerated by plate counting and compared to bacterial titers of moribund mice at 66 h post-infection from the initial Tn-seq transposon library challenge. The limit of detection of bacterial enumeration from host tissue is indicated by a dashed line. Tissue counts below the limit of detection were set to the limit of detection for statistical analysis. Statistical significance was calculated by Two-Way ANOVA followed by Bonferroni post-test (^**^P < 0.01;^***^P < 0.001; ns, not significant).

To characterize the kinetics of disseminated spread from the lung to other tissues, a time course analysis was conducted. Groups of three C57BL/6J female albino mice were infected with 10^4.62^ CFU of the Tn-seq library, and then lungs, liver and spleen were isolated at 6, 12, and 24 h post-infection and bacterial titers were enumerated from each organ. By 6 h post-infection, bacteria were cultured from the lungs with 5.5-fold higher titers than the original instilled amount and grew exponentially throughout the course of infection (Figure 3), consistent with growth patterns observed by optical diagnostic imaging (Figure 1A). At 6 h post-infection, 10^4.87^ CFU were also present in the liver, which were similar titers to those found in the lungs (Figure 3), confirming the indications of the Tn-seq data that hepatic spread is early and at high titer. Colonization of the spleen by 12 h post-infection was at or below the limit of detection (100 CFU), where pronounced splenic colonization only occurred 24 h post-infection (Figure 3), suggesting that the enhanced loss of Tn-seq library diversity in the spleen relative to the lung is associated with later spread from the lung, subsequent to the effects of selective pressure in the lung. Thus, *B. pseudomallei* colonizes host lungs very early during infection and persists at this site through the onset of acute respiratory disease. Similarly, the bacterium can spread from the lungs at high titer to colonize the host liver very early following infection but bacterial titers at this site remain constant until late in infection. This finding corroborates a unique observation made from the Tn-seq data set, shedding new light on *B. pseudomallei* dissemination.

### Identification of virulence determinants required for *B. pseudomallei* colonization of host lungs

**Figure 4:**
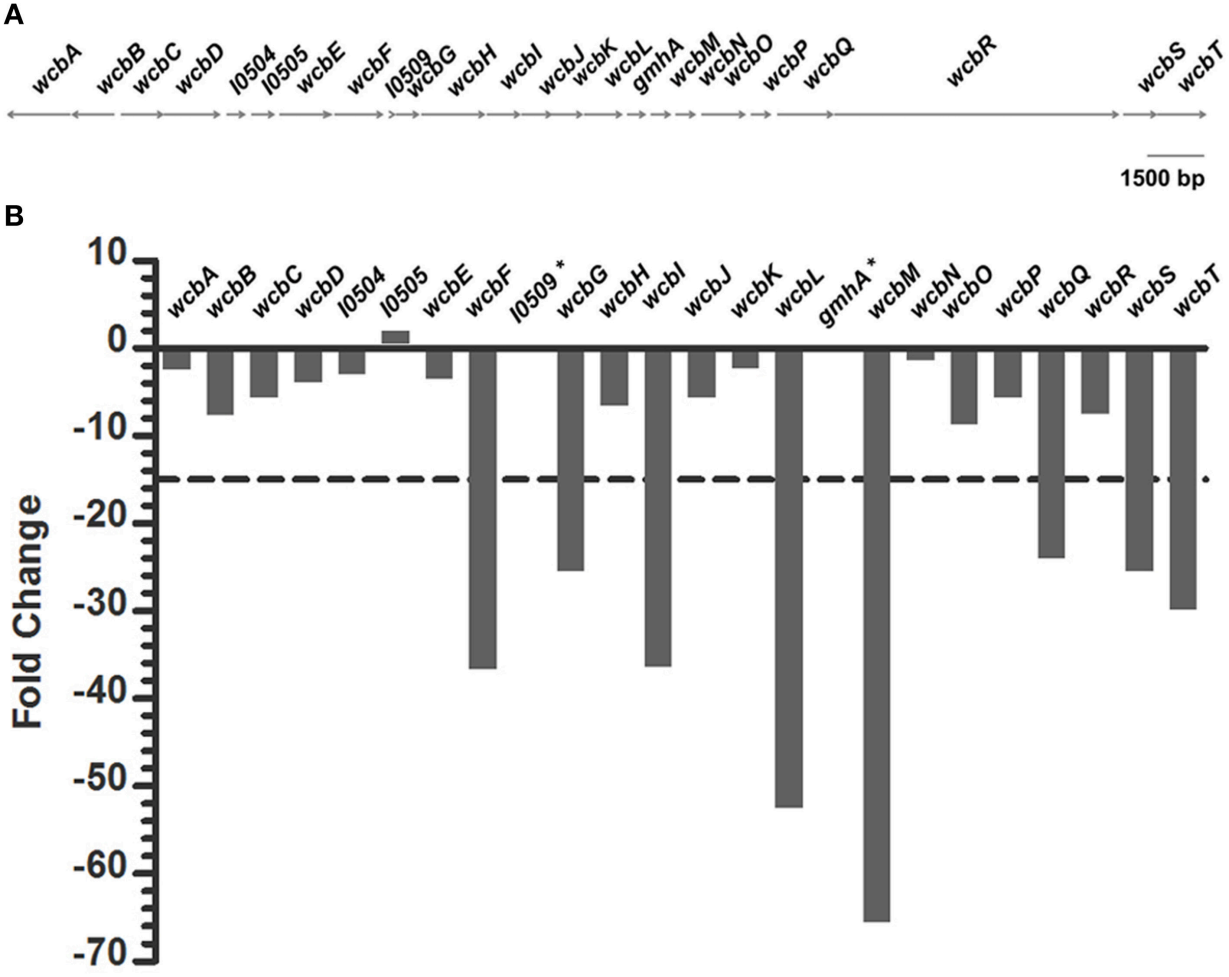
**Analysis of variation within capsular polysaccharide operon genes required for lung colonization by Tn-seq**. **(A)** Scale representation of the capsular polysaccharide genetic locus. **(B)** Variation in fold-change of capsule genes by Tn-seq analysis of the lung data set relative to the input pool. The average fold reduction across all capsule genes was 14.92-fold (dashed horizontal line). ^*^Denotes genes not covered in the input library.

We initially focused our analysis of the Tn-seq data set on identifying virulence determinants required by *B. pseudomallei* to colonize the lungs of mammalian hosts, of which 1455 genes were required to colonize the murine lung at a 3-fold relative abundance cutoff ratio relative to the input pool (Figure 2B). As a refined stringency to focus our identification of key virulence determinants, we made use of a targeted analysis of the capsular polysaccharide biosynthetic cluster (CPS or CPS I, Reckseidler-Zenteno et al., 2009). We have previously demonstrated that a capsule mutant was attenuated 6.8-fold relative to the isogenic parent strain in the IMIT model, but that this level of attenuation was not statistically significant (Gutierrez et al., 2015). We therefore performed a detailed analysis of the Tn-seq lung results for CPS biosynthetic cluster as a benchmark genetic system which exhibits a marginal level of attenuation by LD_50_ analysis. We found that while the majority of CPS genes were reduced in the lung relative to the input pool, pronounced variation in degree of response was observed across the genetic cluster (Figure 4). This variation was suggestive of bottlenecking effects which are possible under the parameters of the current study, designed for a biologically relevant infection while achieving saturating library coverage. While variation was observed across the CPS locus, the locus itself exhibited an average 14.92-fold reduction in mutant prevalence for the lung vs. input pool. Thus, to further analyze the Tn-seq lung data set, we set a more stringent fold-change cut off of 15-fold, reflecting the magnitude of the average fold gene reduction of the CPS locus. Further, we decided to identify clusters of genes which meet this 15-fold cutoff to mitigate the impact which bottlenecking might have on dataset variation at the individual gene level (Figure 4).

**Figure 5:**
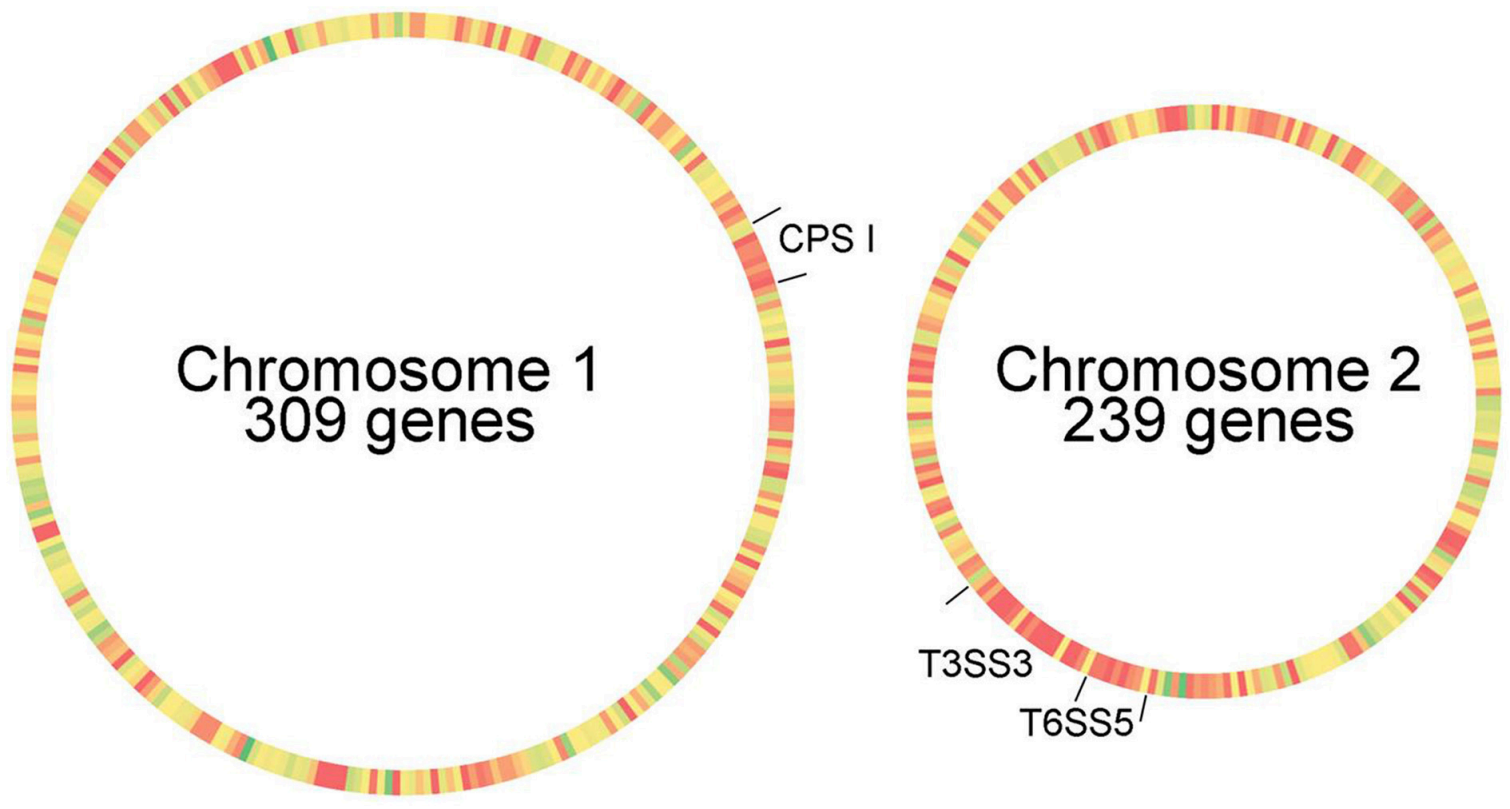
**Proximity heat map of genes required for respiratory melioidosis**. The Tn-seq lung data set was filtered for genes required by *B. pseudomallei* at a 15-fold reduction cut off relative to the input pool (548 genes). Genes were sorted for their arrangement on the two circular chromosomes of the *B. pseudomallei* genome, with similar distribution density found on the larger chromosome 1 (309 genes) and smaller chromosome 2 (239 genes). The heat map provides graphical cluster analysis of gene hits located in close proximity (red = 1 gene distance) or distal hits (green >70 gene neighbor distance). The three largest genetic clusters of hits separated by no more than 4 genes per hit are indicated: capsular polysaccharide (CPS I), Type 3 Secretion System cluster 3 (T3SS3), and Type 6 Secretion System cluster 5 (T6SS5).

A total of 548 genes underwent selective pressure in the lung at a 15-fold cutoff relative to the input pool. These genes were heat mapped for nearest neighbor relationships (Figure 5) to identify genetic clusters with consecutive hits of no more than a 4 gene distance from the previous hit. The three genetic loci which provided the largest assemblage of genes meeting these criteria included: (i) CPS (8 genes), (ii) Type 6 Secretion System cluster 5 (T6SS5; 8 genes), and (iii) Type 3 Secretion System cluster 3 (T3SS3; 7+5 genes) (Table 2). There are six distinct T6SS clusters in the *B. pseudomallei* genome, and the T6SS5 is identified as cluster 5 by the NCBI database (Shalom et al., 2007), and this same cluster is identified as cluster 1 in *Burkholderia mallei* (Schell et al., 2007). Numerous additional small genetic clusters may provide important contributions to the fitness of *B. pseudomallei* in the lung; however, these data indicate that the three prominent genetic systems contributing to *B. pseudomallei* lung pathogenesis are CPS, T3SS3, and T6SS5. Given that we have previously characterized the contribution of CPS and T3SS3 to respiratory melioidosis using the IMIT model (Gutierrez et al., 2015), we decided to address what impact the T6SS5 system has on the virulence of *B. pseudomallei*.

**Table 2:** **Identification of prominent genetic loci required for pulmonary disease**.

**Feature ID**	**Fold change**	**Distance to previous hit**	**Feature ID**	**Fold change**	**Distance to previous hit**
**T3SS3**			**T6SS5**		
*bprA*	−70.3781		BP1026B_II1587	−83.2298	
*bipC*	−35.0191	1	hcp−5	−367.19	4
*bipB*	−15.6668	1	tssF−5	−22.3609	3
*bicA*	−15.2996	1	clpV−5	−23.6294	1
*bsaZ*	−56.991	1	tagB−5	−21.4194	3
*spaP*	−15.6396	3	tagC−5	−22.6434	1
*bsaV*	−26.665	1	tssK−5	−31.7466	2
*bsaO*	−82.8959	7	tssL−5	−111.177	2
BP1026B_II1643	−34.0416	2	**Capsule**		
BP1026B_II1644	−59.6684	1	wcbF	−36.6525	
BP1026B_II1645	−40.3909	1	wcbG	−25.4066	2
BP1026B_II1646	−33.6591	1	wcbI	−36.413	3
			wcbL	−52.5586	3
			wcbM	−65.6182	2
			wcbQ	−24.1121	4
			wcbS	−25.4738	2
			wcbT	−29.8059	1

Only a single gene met the 15-fold cutoff criterion in the liver (thioredoxin: -16.2-fold liver:input), suggesting that the liver does not offer a strong selective pressure against *B. pseudomallei* relative to that of the lung. Conversely, 2255 genes met this more stringent 15-fold cutoff criterion in the spleen, thus this criterion did not effectively reduce the number of hits in the spleen from the 2862 hits at a 3-fold cutoff criterion. Due to the late spread to the spleen and the large number of gene hits, we interpret that the colonization of the spleen in this current model system is a bottle-necked event that does not facilitate a biologically relevant data interpretation. This study therefore focused on validating the results from the lung dataset, specifically the identification of capsule, T3SS3 and T6SS5 as the largest genetic loci contributing to respiratory melioidosis.

### T6SS5 mutant is not attenuated in the IMIT model

**Figure 6:**
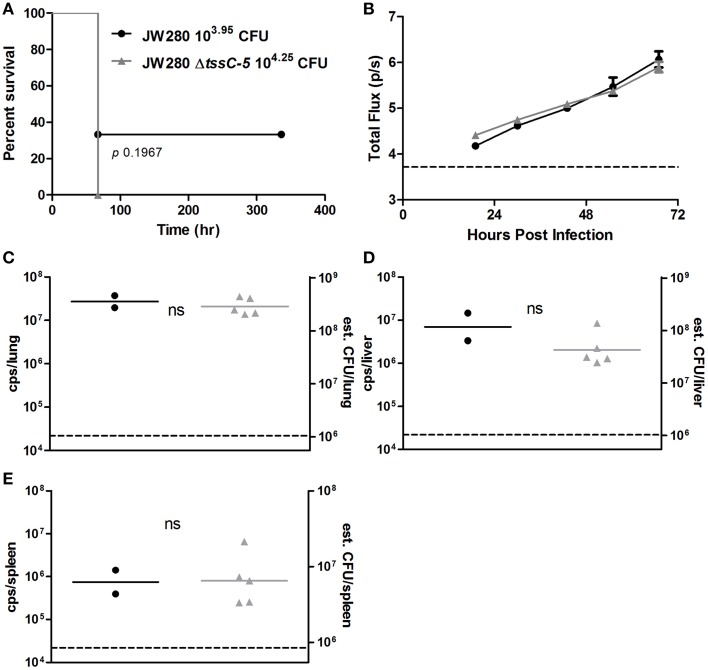
***In vivo* characterization of a T6SS mutant**. **(A)** Survival curve of C57BL/6 female albino mice by IMIT infected with either JW280 or JW280 Δ*tssC-5*. **(B)** *In vivo* growth of *B. pseudomallei* strains JW280 and JW280 Δ*tssC-5* infecting C57BL/6 mice, where bioluminescence was monitored twice per day and thoracic cavity total flux (p/s) was collected from region of interest measurements. The technical 95% limit of detection is indicated by a horizontal dashed line. Bacterial titers in the lungs **(C)**, liver **(D)**, and spleen **(E)** of C57BL/6 mice infected with either JW280 or JW280 Δ*tssC-5* were enumerated by bioluminescence measurements of *ex vivo* tissues (counts per second [cps]/tissue), as described (Lawrenz et al., 2014). Statistical significance was calculated using the Student's *T*-test (ns: not significant).

We previously calculated the LD_50_ of a *B. pseudomallei* luminescent strain JW280 to be 10^3.87^ CFU, a T3SS3 translocation-deficient mutant, JW280 Δ*sctU*_*Bp*3_ to be 10^6.19^ CFU and a CPS operon mutant to be 10^4.57^ (Gutierrez et al., 2015). While CPS, T3SS3, and T6SS5 all contributed to *B. pseudomallei* pulmonary fitness by the Tn-seq screen, single strain challenge with a CPS mutant did not reveal an attenuation by LD_50_, whereas a T3SS3 mutant was significantly attenuated >200-fold (Gutierrez et al., 2015). We therefore challenged C57BL/6J albino female mice with 10^3.95^ CFU of the JW280 luminescent strain and 10^4.25^ CFU of the JW280 Δ*tssC-5* luminescent T6SS5 mutant strain to investigate the role of T6SS5 in respiratory melioidosis. Infection with the parent JW280 strain close to its LD_50_ resulted in 67% mortality (Figure 6A), whereas infection with the luminescent T6SS5 mutant was 100% lethal with the 10^4.25^ CFU dose, which is 0.52-log above the parent LD_50_. Thus, the LD_50_ of the *tssC-5* mutant is < 10^4.25^ CFU, which was not significantly attenuated by Log-rank survival analysis relative to the parent control (Figure 6A). Therefore, like the CPS mutant, the T6SS5 mutant is not attenuated in single strain respiratory model challenge. Optical diagnostic imaging was used to monitor bacterial proliferation in the lung of lethally infected mice, and by 19 h post-infection, both the parent and the T6SS5 mutant were detectable by bioluminescent imaging, however the bioluminescent signal intensity increased at similar rates in the thoracic cavity (Figure 6B). While the T6SS5 mutant appeared to be slightly less fit than parent strain in proliferation rate, this difference was not significant. We characterized the bacterial tissue burden in moribund mice and found no significant difference between the T6SS5 and parent bacterial titers in the lungs, liver and spleen (Figures 6C–E). Thus, although our Tn-seq analysis revealed that the T6SS5 cluster is required for the full fitness of *B. pseudomallei* in the lung, single strain pulmonary challenge studies did not support this role for T6SS5.

### A *B. pseudomallei* T6SS5 mutant is attenuated in competition studies

**Figure 7:**
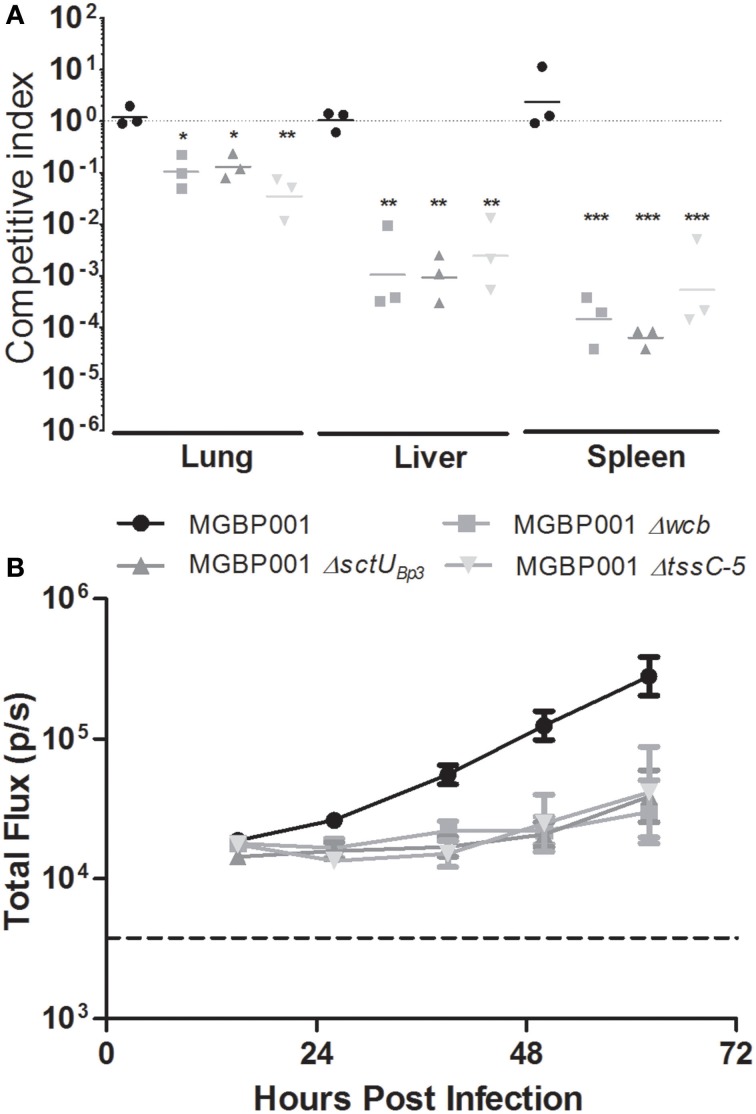
***Burkholderia pseudomallei* major virulence determinants are attenuated in competition assays**. **(A)** Competitive index of luminescent, Gm-marked parent strain (MGBP001), capsular polysaccharide deletion mutant (MGBP001 Δ*wcb*) and T3SS (MGBP001 Δ*sctU*_*Bp*3_) and T6SS5 (MGBP001 Δ*tssC-5*) mutants compared to Gm-sensitive, non-luminescent strain, DD503. Competitive index was calculated as the output ratio of mutant to parent divided by the input ratio of mutant to parent, having differentially plated lung, liver and spleen homogenates on agar plates with or without gentamicin selection. The horizontal dashed line indicates a competitive index of 1. Statistical significance was calculated using One-Way ANOVA followed by Tukey's Multiple Comparison Test (^*^P < 0.01; ^**^P < 0.001; ^***^P < 0.0001).**(B)** In *vivo* growth of MGBP001, MGBP001 Δ*wcb*, MGBP001 ΔsctU_*Bp*3_, MGBP001 Δ*tssC-5* in the lungs was monitored twice daily using luminescence as a read out for bacterial replication, and total flux (p/s) measurements collected from regions of interest centered on the thoracic cavity. The horizontal dashed line indicates the 95% technical limit of detection.

Given that Tn-seq is a genome-wide competition study, and initial validation of our Tn-seq results using traditional LD_50_ estimations did not identify attenuation for the *tssC-5* mutant, we decided to investigate whether this T6SS5 mutant is attenuated by direct competition studies. We generated a novel gentamicin-marked P_tolC_*luxCDABE* bioluminescence reporter strain, MGBP001, which we also generated for the CPS, T3SS3, and T6SS5 mutants, and used these in competition studies against gentamicin-sensitive, non-luminescent, DD503. Accordingly, four groups of three C57BL/6J albino female mice were inoculated with ~10^4.0^ *B. pseudomallei* at a 1:1 ratio of DD503 combined with either: (i) MGBP001, (ii) MGBP001 Δ*wcb* (CPS^−^), (iii) MGBP001 Δ*sctU*_*Bp*3_ (T3SS3-), or (iv) MGBP001 Δ*tssC-5* (T6SS5-). Infections persisted for 67 h (late stage disease) before lungs, liver and spleen were collected and homogenates differentially plated on agar to identify proportions of gentamicin resistant and sensitive strains. The MGBP001 parent strain exhibited a competition index which was not significantly different from a ratio of 1.0 (Figure 7A), indicating that DD503 and MGBP001 shared the same virulence potential in all tissues, suggesting that neither the luminescence operon nor gentamicin resistance impact the virulence of the MGBP001 *B. pseudomallei* parent strain. Interestingly, all three mutant strains exhibited significantly reduced competition indices in the lungs, liver and spleen of mice relative to the non-luminescent DD503 competition partner (Figure 7A). Furthermore, optical diagnostic imaging of the thoracic cavity demonstrated that the parent MGBP001 strain proliferated within the lung (Figure 7B), consistent with the pulmonary colonization observed by the JW280 strain (Gutierrez et al., 2015). Imaging was able to successfully monitor the decreased fitness of the three mutants in the lung during the course of infection (Figure 7B), with significant decreases in thoracic cavity bioluminescence for all but the capsule mutant by 39 h, and for all strains by 50 h. Thus, optical diagnostic imaging successfully provides an early indication of fitness defects *in vivo* during competition studies. Therefore, CPS, T3SS3, and T6SS5 are all critical virulence determinants to support the fitness of *B. pseudomallei* in the lung, but importantly, attenuation of these virulence systems by single strain challenge studies has only been able to identify a role for T3SS3. We propose that competition analyses, including Tn-seq, provide a higher resolution assay by which to assess the role of *B. pseudomallei* virulence determinants in the lung, and that our current Tn-seq screen successfully identified CPS, T3SS3, and T6SS5 as the largest genetic loci required to facilitate respiratory melioidosis.

## Discussion

In this study, we performed Tn-seq analysis to identify virulence determinants required to cause respiratory disease in a mouse model of lung-specific melioidosis. The primary design criterion of our screen was achieving the balance of saturating transposon mutagenesis while also limiting the challenge dose to allow for a biologically relevant disease progression. Previous transposon-directed insertion site sequencing and Tn-seq libraries have been generated in the *Burkholderia pseudomallei* strain K96243 (Moule et al., 2014) and its close relative *Burkholderia thailandensis* (Baugh et al., 2013; Gallagher et al., 2013) to identify essential genes required for survival of these pathogens *in vitro*. Importantly, these *in vitro* screens allowed for much larger library sizes to identify essential genes which were not amenable to transposon mutagenesis, which for *B. pseudomallei* was estimated to be 8.0% of the genome (Moule et al., 2014). In spite of limiting library size in our study to allow for infection studies, we achieved coverage of 88% of the 6141 estimated genes, where the theoretical maximum coverage is 92% based on estimates of number for essential genes. While our Tn-seq library was not designed to identify essential genes, we performed a ortholog search of the *B. pseudomallei* K96243 TraDIS-identified essential genes (Moule et al., 2014), and of the 483 orthologs in the 1026b genome, 53 genes were similarly identified with no reads in our Tn-seq library. An additional 202 K96243 essential orthologs were low read hits in our Tn-seq data set at one standard deviation below the log transformed mean read density. Thus, our Tn-seq data set confirms ~53% of the essential genes in the K96243 TraDIS essential gene list as confirmed essential genes in 1026b orthologs. The remaining half of the predicted essential genes were successfully read in our Tn-seq dataset, and we propose that these are not essential genes in 1026b, including such systems as Type 3 Secretion System cluster 1 (T3SS1) and three of the Type 6 Secretion Systems (T6SS2, T6SS4, and T6SS5), notably including T6SS5 which was a subject of investigation in these studies. Thus, consistent with the wider body of literature, some of the genes previously reported as essential may not be so, notably including the report that *amrA* and *amrB* are essential genes (Moule et al., 2014); while we do not read these genes in our Tn-seq dataset due to the fact that they have been deleted from DD503-linage strains, these are clearly not essential genes due to the availability of strains lacking these genes (Moore et al., 1999). These findings may suggest that the essential gene repertoire of *B. pseudomallei* is smaller than previously reported, and may reflect contributions from differences in strain (K96243 vs. 1026b lineage), transposon (Tn5 vs. Marineer) and other potential culturing techniques.

An unexpected result of our Tn-seq dataset was the revelation that *B. pseudomallei* is capable of reaching the liver very early in the course of infection, which was subsequently validated by a time course infection study. These findings were facilitated by using IMIT instillation which has been reported to efficiently deliver < 98% of an instilled dose directly into the lung (Lawrenz et al., 2014). Within 6 h of delivery directly into the lung by IMIT instillation, *B. pseudomallei* not only replicates in the lung, but also spreads to the liver at numbers similar to that cultured from the lung. Interestingly, few genes were critical for *B. pseudomallei* to maintain a presence in the liver, relative to the lung, suggesting that the lung does not persistently seed the liver over the course of infection, nor is the liver a source of a strong selective pressure to *B. pseudomallei*. Furthermore, *B. pseudomallei* does not appear to replicate in the liver until late in the infection, suggesting that the systemic fitness of the host may impact the replication potential of *B. pseudomallei* in the liver, again given that Tn-seq results do not support the possibility that the late infection increase in hepatic burden results from spread from the lung. It is noteworthy that in other respiratory models, the liver becomes colonized at later time points; for instance, aerosol delivery of *B. pseudomallei* did not result in detection of bacteria in the liver until 3 days post-infection (Tan et al., 2008). Thus, the observation of rapid dissemination from the lung to the liver may be a unique feature of the IMIT model, as is the prominent moribund septicemia which is not observed in other respiratory melioidosis models. Future work will be required to understand the role of the liver as a replicative niche for *B. pseudomallei* during respiratory melioidosis.

We developed a high specificity filter to prioritize follow-up analysis of the Tn-seq results of the lung. The selection criteria was benchmarked to the capsule polysaccharide genetic locus which we previously demonstrated was not a critical virulence determinant in the IMIT model of respiratory melioidosis, yet was attenuated 6.8-fold relative to parent (Gutierrez et al., 2015). Thus, our criteria was chosen to capture all virulence systems, including those which may not be significantly required by LD_50_ analysis. Our screen identified 548 genes, 8.7% of the genome's predicted ORFs, to be required by the bacterium for mammalian lung colonization, and of these, 32% (175 ORFs) accounted for hypothetical proteins, suggesting that additional novel virulence systems may participate in mediating fitness within the mammalian lung. Using cluster analysis to further prioritize the Tn-seq data set, we identified the three dominant genetic loci contributing to respiratory melioidosis as CPS, T3SS3, and T6SS5. Interestingly, these systems have been previously identified as virulence systems in systemic animal models (Reckseidler et al., 2001; Warawa and Woods, 2005; Burtnick et al., 2011); however, other virulence determinants described as important in systemic disease models did not meet our selection criteria, including the lipopolysaccharide (LPS) biosynthetic locus which had just two genes meeting our 15-fold selection criterion. Thus, virulence determinants required to support systemic infection may not be the same as those required in the lung. Our Tn-seq analysis was notably biased to the identification of large genetic systems which contribute to respiratory melioidosis, and it is therefore likely that additional smaller genetic loci have also been identified by this Tn-seq dataset, which will be the subject of future investigation.

Most *B. pseudomallei* virulence factors have been characterized primarily in systemic infection models, including intravenous, subcutaneous, and intraperitoneal inoculation. Indeed, the three major virulence systems targeted from our Tn-seq screen have been well studied as major contributors to disease in hamster and mouse systemic intraperitoneal infection models. The capsular polysaccharide has been characterized by several groups as providing one of the most significant contributions to the systemic disease potential of *B. pseudomallei*, with capsule mutants being attenuated ~5 log in these infection models (Atkins et al., 2002; Reckseidler-Zenteno et al., 2005). The significant role for capsule in *B. pseudomallei* systemic disease is consistent with a demonstrated role of this virulence factor in resisting complement opsonization of the bacteria (Reckseidler-Zenteno et al., 2005), and therefore likely a critical virulence factor for traffic between tissues, suggestive of *B. pseudomallei* spread as an extracellular pathogen. Interestingly, the capsule mutant does not appear to have a major role in the respiratory system of mice by single strain challenge, with attenuation levels of < 2 log by intranasal delivery and < 1 log by IMIT instillation (Warawa et al., 2009; Gutierrez et al., 2015). The T3SS3 system is known to importantly allow for escape of *B. pseudomallei* from phagosomes to facilitate rapid replication in the cytoplasm of phagocytes (Stevens et al., 2002), and the ubiquitous requirement for T3SS3 in both systemic (Warawa and Woods, 2005) and respiratory disease models (Gutierrez et al., 2015) suggests that the intracellular lifestyle of *B. pseudomallei* is critical in all host tissues. Indeed, T3SS3 is the only virulence system examined thus far in the IMIT instillation method which exhibits significant attenuation by single strain challenge, suggesting that this virulence system is one of the most critical systems for both respiratory and systemic disease. The primary function of the T6SS5 system is not well described, though there is evidence that T6SS5 supports the intracellular lifestyle of *B. pseudomallei* and cell-to cell spread (Pilatz et al., 2006; Burtnick et al., 2011). T6SS5 system mutants are attenuated >3 log in a systemic hamster intraperitoneal model (Burtnick et al., 2011) and are attenuated ~2–3 log in intranasal murine models (Pilatz et al., 2006; Hopf et al., 2014). In this current study, the role for T6SS5 in respiratory melioidosis in single strain challenges using IMIT delivery does not appear to suggest a prominent role given that we were unable to demonstrate significant attenuation in single strain challenge studies, which is in contrast to what has been previously reported for the intranasal models. We have previously demonstrated that intranasal inoculation of *B. pseudomallei* in mice results in a moribund disease state primarily associated with the nasal cavity rather than the systemic disease state observed following IMIT inoculation (Gutierrez et al., 2015). Thus, T6SS5 may be an important factor for nasal cavity colonization, but does not play the same critical role in the lung as seen for the T3SS3 system. In spite of the inability of capsule and T6SS5 mutants to demonstrate T3SS3-like significant attenuation by single strain challenges, they are clearly virulence determiants when assessed in competition studies.

A technical caveat of these studies relates to the use of a cloning strain of *B. pseudomallei* to perform the Tn-seq screen. The Tn-seq library was developed using the JW280 bioluminescent strain, which was derived from the aminoglycoside-sensitive strain DD503, which is itself derived from the 1026b clinical isolate. We have previously demonstrated that the introduction of the *luxCDABE* bioluminescent operon into the *B. pseudomallei* genome did not impact the virulence of the bacteria (Warawa et al., 2011), which was validated in this study by the finding that introduction of both *luxCDABE* and the gentamicin resistance marker (MGBP001) did not impact the competition index relative to the parent strain, DD503. It is important to note that the deletion of the AmrAB-OprA efflux pump in the DD503 strain may have an impact on virulence of *B. pseudomallei* and thus impact which genes are required for lung fitness in that specific strain background.

A key finding of our studies was the ability of competition studies to provide a higher resolution identification of virulence factors in the fitness of *B. pseudomallei* in the lung than single strain challenges/LD_50_ analyses. Similar findings have been made for other disease model systems, where competition index provides a higher resolution methodology for defining the contribution of virulence genes to a pathogen's fitness in animal model systems (Auerbuch et al., 2001; Logsdon and Mecsas, 2003; Yang et al., 2012). We had previously reported that a CPS mutant was not significantly attenuated by LD_50_ in the IMIT model (Gutierrez et al., 2015), but presently that CPS was significantly attenuated by competition study in the IMIT model. Similarly, we were unable to validate a significant attenuation for a T6SS5 mutant by single strain challenge, but identified a significant attenuation by competition index. The Tn-seq screen itself is a genome wide competition assay, thus our competition studies were able to successfully recapitulate the phenotypes we identified from the Tn-seq screen results, whereas single strain challenges did not have the resolution to defined roles in respiratory melioidosis to either capsular polysaccharide or T6SS5. These observations indicate that competition studies have a higher resolution to ascribe roles in virulence to both CPS and T6SS5 in respiratory melioidosis, and thus far, T3SS3 is the sole virulence determinant which exhibits attenuation by LD_50_ in the IMIT respiratory melioidosis model (Gutierrez et al., 2015). As an added feature of our competition assay, we have included a unique optical imaging approach which is further capable of detecting significant fitness defects in competition studies as early as 39 h post-infection.

An additional aspect of these studies worth highlighting is the finding that significant phenotypes were able to be resolved using small group sizes of animals. The three Rs of responsible animal research include: (1) replacement, (2) reduction, and (3) refinement; it is notable that reduction in group size was achievable through the methods we employed. By using groups of three mice inoculated by IMIT inoculation and infection monitoring by bioluminescence, we are successful in very targeted, efficient delivery of reagents directly into the lung (Lawrenz et al., 2014), which reduces variation in study metrics. This was particularly apparent in the demonstrated ability to synchronize infections and achieve excellent bacterial burden analysis throughout our studies. Most host-pathogen interactions are successfully studied by triplicate analysis, and we have demonstrated that *n* = 3 studies can be successfully carried out using whole animal host-pathogen interaction studies as well. This is consistent also with the previous use of small groups of animals (3–4 animals) to perform pooled *in vivo* Tn-seq analysis, and such study design has successfully resolved the detection of virulence systems for other bacterial pathogens (Skurnik et al., 2013a,b; Wang et al., 2014).

In summary, we provide the first phenotypic screen for *B. pseudomallei* virulence factor function in the mammalian lung, and have identified the largest critical genetic systems as CPS, T3SS3, and T6SS5. While previously identified as virulence systems, this study represents the first comparative study to validate their importance to respiratory melioidosis. These virulence systems therefore represent excellent targets for the future development of therapeutics against this Tier 1 Select Agent pathogen.

## Author contributions

All authors contributed to experimental design, conducting studies, and composing the manuscript (MG, DY, JW).

### Conflict of interest statement

The authors declare that the research was conducted in the absence of any commercial or financial relationships that could be construed as a potential conflict of interest.
